# Effective coverage of primary care services in eight high-mortality countries

**DOI:** 10.1136/bmjgh-2017-000424

**Published:** 2017-09-04

**Authors:** Hannah H Leslie, Address Malata, Youssoupha Ndiaye, Margaret E Kruk

**Affiliations:** 1 Department of Global Health and Population, Harvard TH Chan School of Public Health, Boston, Massachusetts, USA; 2 Kamuzu College of Nursing, Malawi University of Science and Technology, Limbe, Southern Region, Malawi; 3 Planning, Research and Statistics, Ministry of Health and Social Action, Dakar, Senegal

**Keywords:** Child Health, Health Systems Evaluation, Maternal Health, Cross-sectional Survey, Health Services Research

## Abstract

**Introduction:**

Measurement of effective coverage (quality-corrected coverage) of essential health services is critical to monitoring progress towards the Sustainable Development Goal for health. We combine facility and household surveys from eight low-income and middle-income countries to examine effective coverage of maternal and child health services.

**Methods:**

We developed indices of essential clinical actions for antenatal care, family planning and care for sick children from existing guidelines and used data from direct observations of clinical visits conducted in Haiti, Kenya, Malawi, Namibia, Rwanda, Senegal, Tanzania and Uganda between 2007 and 2015 to measure quality of care delivered. We calculated healthcare coverage for each service from nationally representative household surveys and combined quality with utilisation estimates at the subnational level to quantify effective coverage.

**Results:**

Health facility and household surveys yielded over 40 000 direct clinical observations and over 100 000 individual reports of healthcare utilisation. Coverage varied between services, with much greater use of any antenatal care than family planning or sick-child care, as well as within countries. Quality of care was poor, with few regions demonstrating more than 60% average performance of basic clinical practices in any service. Effective coverage across all eight countries averaged 28% for antenatal care, 26% for family planning and 21% for sick-child care. Coverage and quality were not strongly correlated at the subnational level; effective coverage varied by as much as 20% between regions within a country.

**Conclusion:**

Effective coverage of three primary care services for women and children in eight countries was substantially lower than crude service coverage due to major deficiencies in care quality. Better performing regions can serve as examples for improvement. Systematic increases in the quality of care delivered—not just utilisation gains—will be necessary to progress towards truly beneficial universal health coverage.

**Key questions**

**What is already known about this topic?**

- Health system contributions to population health require both appropriate utilisation of services by those in need and delivery of high-quality care, which can be jointly expressed as effective coverage.

**What are the new findings?**

- Quality of health services in antenatal care, family planning and sick-child care was consistently poor across eight low-income and middle-income countries: adherence to evidence-based guidelines was under 50% in many cases.
- Effective coverage is considerably lower than crude coverage for all three services due to poor quality; variation within countries suggests improvement is possible based on existing best performers.

**Recommendations for policy**

- Policymakers should devote greater attention to bolstering health service quality, particularly for services that have achieved relatively high utilisation, to optimise population health outcomes.
- Coordinated measurement of health service need, utilisation and quality is needed in order to monitor and incentivise progress towards universal health coverage.

## Introduction

The transition in 2015 from the Millennium Development Goals (MDG) to the Sustainable Development Goals (SDG) solidified a shift in global health from service-specific targets to broader health system goals.^1 2^ SDG goal 3 explicitly calls for the achievement of universal health coverage, with quality of healthcare services as an integral component.^2^ This evolution reflects the growing recognition that provision of a range of quality services is central to health systems delivering benefits to the population they serve.^3 4^

Expanding the focus beyond access to quality has led to an increased interest in measuring effective coverage, defined as the fraction of potential health gain actually delivered through the health system to the population in need.^5^ The WHO and World Bank have identified measuring and improving effective coverage as critical to achieving universal health coverage.^6^ Health systems deliver optimum health gains when those in need both access services and receive high-quality care. One approach to estimating effective coverage is the product of utilisation and service quality conditional on need for the service.^7^ It is a flexible construct that can be applied to specific elements of a given clinical service, the entirety of a service or a combination of essential health system services.^8^

We focus here on three essential services within primary care: antenatal care (ANC), family planning and care for sick children under 5. Successful delivery of each service is essential to achieving the SDG targets on maternal, neonatal and under-5 mortality,^9^ but research to date has identified substantial variation in the clinical quality of these services in low-income and middle-income countries.^10^ While these services are far from the entirety of primary care, their widespread provision and the existence of comparable crossnational measurement of their utilisation and quality make them useful tracer services for the measurement of effective primary care. Researchers and policymakers have called for better measures of primary care performance in particular to monitor health system strengthening efforts.^11^ As demonstrated by disproportionate progress towards MDG targets relative to other health system indicators,^12^ crossnational measurement may be an important tool in encouraging improved performance.

While crude coverage can typically be estimated from household survey data, assessing effective coverage depends on valid and appropriate measures of quality,^7^ which require clear conceptualisation and sufficient data to achieve. Quality measures are commonly organised into three domains: structure, process and outcomes.^13^ Each domain provides distinct insights into health service delivery: the basic input and capacity necessary to provide care, the actual clinical content of care, and the patient outcome thereafter based on both the quality of care and factors unrelated to the health system. Following Tanahashi’s original health service coverage framework,^14^ research describing coverage combined with measures of structural quality frequently refers to accessible coverage, a precursor to effective coverage.^15^ We review prior research related to effective coverage of ANC, family planning and care for sick children.

Of the three services, ANC has been the subject of most research, with a range of effective coverage studies from subnational assessment to crosscountry comparisons.^15–23^ These studies uniformly measure effective coverage based on receipt of essential services such as blood pressure measurement or blood testing during ANC. For example, studies in Mexico have found that most states reach between half and 80% of women with appropriate ANC,^18^ with greater effective coverage for women with insurance,^22^ while studies in several low-income countries have found that fewer than 15% of women received a minimum set of essential services during pregnancy.^19 21 23^

Research on effective coverage of family planning and sick-child care is much less extensive, with a focus on structural measures such as facility readiness.^16^ Even these measures suggest considerable quality shortfalls: a recent study in Kenya found a drop of 28% in family planning coverage when considering facility readiness. Studies of sick-child care have identified gaps in both access and quality, with effective coverage of acute respiratory illness estimated as only 41% in Kenya and 60% in Mexico.^8 16^ An innovative study of malaria in sub-Saharan Africa combined maternal report of treatment source with studies of treatment type to calculate expected cure rates; the findings suggest an average effective coverage of only 40%, ranging from 7% in Somalia to 71% in Botswana.^24^ Across all services and settings, the findings point to dramatic gaps in population health coverage once quality of care is accounted for.

Out of this body of evidence, two studies discuss the creation of aggregate measures. Nguhiu *et al* 16 present the average of effective coverage for maternal and child health services in Kenya, weighted by population need for the service. They find that aggregate effective coverage is increasing in Kenya, with some suggestion that inequalities between wealthy and poor populations are decreasing. Lozano *et al* 8 explored multiple weighting methods to calculate a metric of health system effective coverage and, finding high concordance across methods, presented the simple average as the most understandable metric. This composite metric usefully identifies the geographical areas of the country most in need of health system improvement.

The findings from the rapidly expanding literature on effective coverage in maternal and child health services demonstrate the need to consider quality in assessing the true population receipt of health services. However, little existing research considers more than a single clinical service or country, limiting generalisability. In this work, we combine nationally representative facility and population survey data from eight countries to evaluate effective coverage of three primary care services at the subnational level. We use quality measures based on directly observed clinical care from facility surveys to adjust coverage measures from population surveys. We compare effective coverage across services, calculate a composite effective coverage metric, and identify gaps in effective coverage both within and between low-income and middle-income countries.

## Methods

### Sample

To calculate the effective coverage of three primary care services across multiple countries, we identified countries with standardised information on population utilisation of care and primary care performance. Countries were eligible for inclusion if a Service Provision Assessment (SPA) survey that included direct observation of primary care services had taken place in the past decade, providing a standardised assessment of the content of care throughout the country. Eight countries met this criterion: Haiti, Kenya, Malawi, Namibia, Senegal, Rwanda, Tanzania and Uganda. To define population in need and utilisation of care, we identified the population-representative survey conducted closest in time to the health facility survey for each country, using Demographic and Health Survey (DHS) household surveys for all countries except Malawi, where we used the Multiple Indicator Cluster Survey (MICS). The Malawi MICS was completed in the same year as the SPA; estimates of utilisation are quite comparable between the 2014 MICS and the 2015–2016 DHS survey. We estimated total population size using the census or census-based projections of total population cited in each DHS or MICS report and applied to these the proportion of the population in the relevant age groups (women 15–49, children under 5) based on the population survey results. Reports for two countries—Malawi and Uganda—did not include total population size; we used the World Development Indicators population estimate for the year of the population survey in these countries.

SPA surveys of the health system are conducted by the DHS Program in collaboration with a national statistics office. Three of the eight countries included in this study elected to conduct a complete census of health facilities: Haiti in 2013, Malawi in 2013 and Namibia in 2009. The SPA in Rwanda in 2007 was a census of public health facilities and large private facilities plus a representative sample of small private facilities. The other four countries (Kenya in 2010, Senegal in 2013–2014, Tanzania in 2015, Uganda in 2007) sampled health facilities from a a master facility list stratified by facility type and subnational region, with deliberate oversampling of hospitals. All SPA surveys included both public and private facilities. The survey includes a facility audit and direct observation of ANC, family planning and curative care for children under 5. Within each sampled health facility, patients presenting for these services were sampled using systematic random sampling; trained observers assessed the care provided according to a checklist of possible provider actions. The data include sampling weights for client visit calculated to account for probability of sampling the client and the facility.

All population and health facility surveys were designed to be representative at a subnational level; we extracted the regional boundaries used in each survey from DHS or the GADM database of global administrative areas for surveys where subnational regions matched national administrative units. Boundaries were not always consistent between the health system and population surveys due to differences in survey design or changes in administrative boundaries over time. We used QGIS mapping software to identify the smallest possible identical spatial areas to which findings could be generalised from both surveys as our units of analyses; we calculated the area of each region and estimated regional population using LandScan (2010) High-Resolution Global Population Data Set.^25^

### Metrics

For each primary care service with available data—ANC, family planning and curative care for children—we used global standards to define the population in need as well as metrics of population coverage of care and technical quality of care delivered. The population in need of ANC was defined as women aged 15–49 with a live birth in the past 2 years; coverage was defined in two ways: attending at least one and the recommended minimum four ANC visits during the most recent pregnancy. Women with contraceptive need were those 15–49 who are married or in a union and wish to space or limit childbearing; women using a modern contraceptive method are considered covered. Children under 5 who had experienced diarrhoea, fever or acute respiratory illness in the prior 2 weeks had a need for curative health services; coverage was calculated as an interaction with a health facility or formal provider. To quantify the number of people in need, we multiplied the relevant population total (eg, women 15–49) by the proportion in need from the household survey (eg, women reporting live birth in the past 2 years).

We defined technical quality of care in each service by identifying key domains of care and the essential clinical actions within each domain from international guidelines.^26–29^ These domains include history, exam and counselling; ANC and sick-child care also include items on testing and management, respectively (full lists for all services are in online supplementary table 1). Example items include the provider asking expectant mothers if they experienced danger signs, providing counselling on the family planning method prescribed and taking a child’s temperature. For each directly observed clinical visit, we calculated the quality score as the per cent of actions completed out of items assessed per country. Actions in follow-up ANC visits were weighted to reflect the number of times they should be performed. For example, provision of tetanus toxoid vaccination contributed one-third of one action since this service should be provided in one of the three follow-up visits.

10.1136/bmjgh-2017-000424.supp1 Supplementary file 1

### Analysis

We summarised population in need, proportion seeking care and average quality of care at the subnational and national levels, weighting individual observations in all population and facility surveys with the appropriate sampling weight. For each subnational region and each country, we multiplied use of healthcare by average quality to yield effective coverage; in the case of ANC we used four visits as the coverage indicator to capture full utilisation. We quantified aggregate primary care coverage, quality and effective coverage measures by averaging across the three services. In this case we used any ANC visit as the coverage indicator for ANC to capture any access to care, matching the other two services.

We report descriptive statistics of the population and health system surveys as well as national summaries for need, coverage, quality and effective coverage. To quantify uncertainty around each estimate, we calculated the standard error (SE) of the mean for proportion in need, proportion seeking care and average quality by country, accounting for repeated sampling by cluster and health facility in population and facility surveys, respectively. Uncertainty estimates were not available for total population sizes; we multiplied the SE of the estimated proportion of the population in need by total population to quantify uncertainty in units of thousands of people. To calculate uncertainty in effective coverage, we used the formula for variance of a product of independent variables,^30^ treating utilisation and quality at the country level as independent due to the separate sampling sources.

We present national summaries of coverage and quality to identify priority deficits in primary care services in study countries. We quantified variation in quality of care by estimating the difference between best and worst effective coverage per country, as well as calculating the intraclass correlation (ICC) by country for each quality metric; we repeated this test on the 5th–95th percentile of the sample by population size to exclude outlying observations. We mapped primary care coverage and primary care quality by subnational region, assessed the correlation between coverage and quality at the subnational level, and plotted coverage versus effective coverage by service.

The original survey implementers obtained ethical approvals for data collection; the Harvard University Human Research Protection Program approved this secondary analysis as exempt from human subjects review.

## Results

The eight countries with health system and population data available to calculate quality-adjusted coverage of primary care are described in table 1, panel A. Gross domestic product per capita ranged from US$255 in Malawi to US$5693 in Namibia, a middle-income country. Populations in these countries confront considerable health burdens, with under-5 mortality and maternal mortality greatly in excess of global targets. Health systems must operate on minimal resources: annual health expenditure per capita is under US$100 in all countries except Namibia.

**Table 1:** Descriptive characteristics of the study

(A) Context of study countries					
	GDP per capita	Health spending per capita	Physicians/100 000 people	Under-5 mortality/1000 births	Maternal mortality/100 000 births
Haiti	$820	$61	25	69	359
Kenya	$1246	$78	20	49	510
Malawi	$255	$29	2	64	634
Namibia	$5693	$499	37	45	265
Rwanda	$638	$52	6	42	290
Senegal	$1067	$50	6	47	315
Tanzania	$695	$52	3	49	398
Uganda	$572	$52	12	55	343

(B) Descriptive statistics of the data set										
Sample sizes										
	Population survey				Health system survey					Regions
	Year	Recently pregnant women*	Women with contra­ceptive need†	Recently ill children<5	Year	Facilities sampled	ANC visits observed	Family planning visits observed	Sick-child care visits observed	
Haiti	2012	3002	6683	3080	2013	905‡	1620	1302	2442	11
Kenya	2008–2009	2410	3867	1955	2010	695	1409	1010	2016	8
Malawi	2013§	7500	11 765	9811	2013	977‡	2068	1499	3329	27
Namibia	2013	1706	5500	1598	2009	411‡	859	983	1544	13
Rwanda	2010	3292	5423	2105	2007	538‡	722	678	1709	5
Senegal	2012–2014	5436	5558	3291	2013–2014	890¶	1211	968	2519	4
Tanzania	2016	4273	5999	2447	2015	1188	4007	1746	4961	30
Uganda	2006	3331	3685	4050	2007	491	779	208	1037	5

Maternal mortality: 2014, modelled; health spending per capita: 2014. Source: Global Development Indicators.

*Women 15–49 who gave birth in the 2 years before the survey.

†Women 15–49 married or in a union who wish to space or limit childbearing.

‡Census or near census of health system.

§Multiple indicator cluster survey.

¶First 2 years of 5-year continuous health system survey.

ANC, antenatal care; GDP, gross domestic product.

Household surveys in these countries included responses from 100 819 of 102 646 (98.2% response rate), yielding 30 950 women with a live birth in the past 2 years, 48 480 women with contraceptive need and 28 337 children with recent symptoms of illness. Health system surveys included 6095 facilities of 6293 sampled (96.8% response rate); these facilities yielded observations of 12 675 ANC visits, 8394 family planning visits and 19 557 curative care for children visits distributed across countries, as shown in panel B of table 1. The median time elapsed between household and facility surveys was 1 year (range 0–4 years).

Table 2 displays the three components of effective coverage for each service and the aggregate at the national level. The population in need of services ranged from approximately 100 000 (pregnant women and sick children in Namibia) to over 5 million (women with contraceptive need in Tanzania). Women with contraceptive need formed the largest subpopulation in each study country. Health service coverage varied considerably between services, with near universal access to any ANC (90% in Haiti to nearly 99% in Malawi), but substantially lower and more varied use of complete ANC, modern contraception and sick-child services, ranging from 35% for contraception in Uganda to 80% for complete ANC in Namibia. Significant differences in average coverage by country emerged, from under 60% in Haiti and Senegal to over 80% in Malawi and Namibia, respectively, the poorest and richest countries in the sample. Across all services and countries, quality of care was lower and less variable than coverage, with clinical adherence to international guidelines ranging from around 0.30 for sick-child care in most countries to a high of 0.65 for ANC in Namibia. Quality was highest in ANC in all countries and lowest in sick-child care in seven of eight countries; average quality across services ranged from 0.37 in Haiti to 0.55 in Namibia. Incomplete access and low quality led to poor effective coverage for each service across all countries, with average effective coverage of 28.3% for ANC, 26.4% for family planning and 20.6% for care for sick children. Effective coverage was highest on average in Namibia (by far the wealthiest country in the sample), at 40.7% across the three services, compared with a low of approximately 19% in Haiti and Senegal.

**Table 2:** Need, coverage, quality and effective coverage of three primary care services

	Haiti	Kenya	Malawi	Namibia	Rwanda	Senegal	Tanzania	Uganda	Total
Estimated need (n±SE, in thousands)									
Women pregnant in the past 2 years	515.2±14.5	2525.8±84.8	1021.0±17.8	113.4±3.0	596.6±11.3	865.7±23.3	3483.7±86.5	2288.1±46.4	11 409.7
Women with contraceptive need	1181.2±16.9	4414.9±77.0	1629.3±17.9	302.7±3.9	970.5±12.3	955.7±16.7	5242.8±77.0	2558.9±40.0	17 256.0
Children under 5 with recent illness	563.8±12.7	2121.8±88.5	1445.3±20.4	99.4±2.8	420.0±11.8	551.8±15.2	2203.8±65.5	3003.9±56.2	10 409.8
Coverage (%±SE)									
ANC—any	90.2%±0.8%	92.6%±1.0%	98.8%±0.2%	95.7%±0.6%	98.2%±0.2%	96.5%±0.4%	97.7%±0.3%	95.5%±0.4%	95.7%
ANC—four visits	64.8%±1.5%	45.0%±1.6%	46.2%±0.8%	79.3%±1.2%	36.0%±1.1%	45.8%±1.3%	48.1%±1.3%	45.2%±1.2%	50.2%
Demand satisfied for modern contraception	46.7%±1.1%	57.9%±1.3%	77.6%±0.6%	84.2%±0.7%	63.4%±0.8%	40.9%±1.5%	56.5%±1.1%	34.9%±1.4%	57.4%
Under-5 care seeking	39.9%±2.3%	50.8%±1.7%	67.3%±0.7%	61.2%±1.7%	41.1%±1.3%	39.1%±1.5%	48.2%±1.8%	74.1%±1.2%	54.4%
Average of three primary care services	58.9%	67.1%	81.2%	80.3%	67.5%	58.8%	67.5%	68.2%	69.2%
Quality (score 0 to 1±SE)									
ANC	0.45±0.01	0.63±0.01	0.50±0.01	0.65±0.02	0.62±0.01	0.57±0.01	0.55±0.01	0.54±0.01	0.57
Family planning	0.36±0.01	0.47±0.01	0.39±0.01	0.44±0.01	0.60±0.02	0.47±0.01	0.45±0.01	0.53±0.04	0.46
Sick-child care	0.30 ±<0.01	0.45±0.01	0.31±0.01	0.54±0.01	0.32±0.01	0.30 ±<0.01	0.32±0.01	0.49±0.01	0.38
Average of three primary care services	0.37	0.52	0.40	0.55	0.52	0.45	0.44	0.52	0.47
Effective coverage									
ANC	29.1%±0.8%	28.5%±1.1%	23.2%±0.6%	51.6%±1.5%	22.5%±0.8%	26.2%±0.8%	21.2%±0.8%	24.3%±0.9%	28.3%
Family planning	16.7%±0.5%	27.3%±0.9%	30.3%±1.0%	37.3%±1.1%	37.9%±1.2%	19.3%±0.8%	25.3%±0.7%	18.4%±1.6%	26.4%
Sick-child care	11.8%±0.4%	22.9%±0.9%	20.7%±0.4%	33.0%±1.1%	13.2%±0.5%	11.7%±0.5%	15.6%±0.6%	36.3%±1.2%	20.6%
Average of three primary care services	19.2%	26.3%	24.7%	40.7%	24.5%	19.0%	22.5%	26.3%	25.4%

ANC, antenatal care.

**Figure 1:**
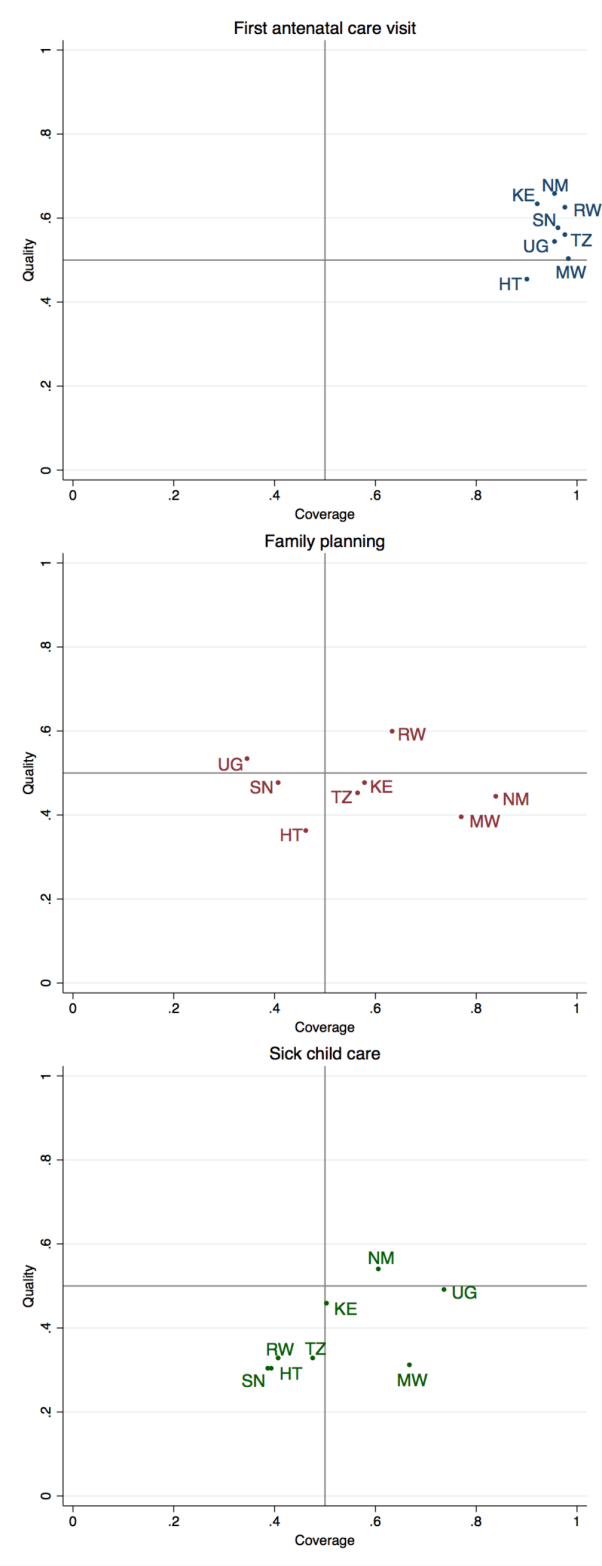
Health system challenges: identifying deficits in coverage and quality of primary care. HT, Haiti; KE, Kenya; MW, Malawi; NM, Namibia; SN, Senegal; TZ, Tanzania; UG, Uganda.

Figure 1 plots health service coverage against quality of care for each country in order to identify health system priorities: countries achieving strong effective coverage would be located in the upper right quadrant. The priority for ANC clearly lies in improving quality of care, with all countries grouped near 1 for access but averaging just over half of basic clinical quality. Family planning services show much greater variability in coverage but consistently inadequate quality; improving clinical quality is a particular priority in countries such as Malawi and Namibia that have attained higher coverage. Children under 5 confront serious gaps in both coverage and quality, with three countries—Rwanda, Haiti and Senegal—achieving especially low coverage and quality of clinical care.

**Figure 2:**
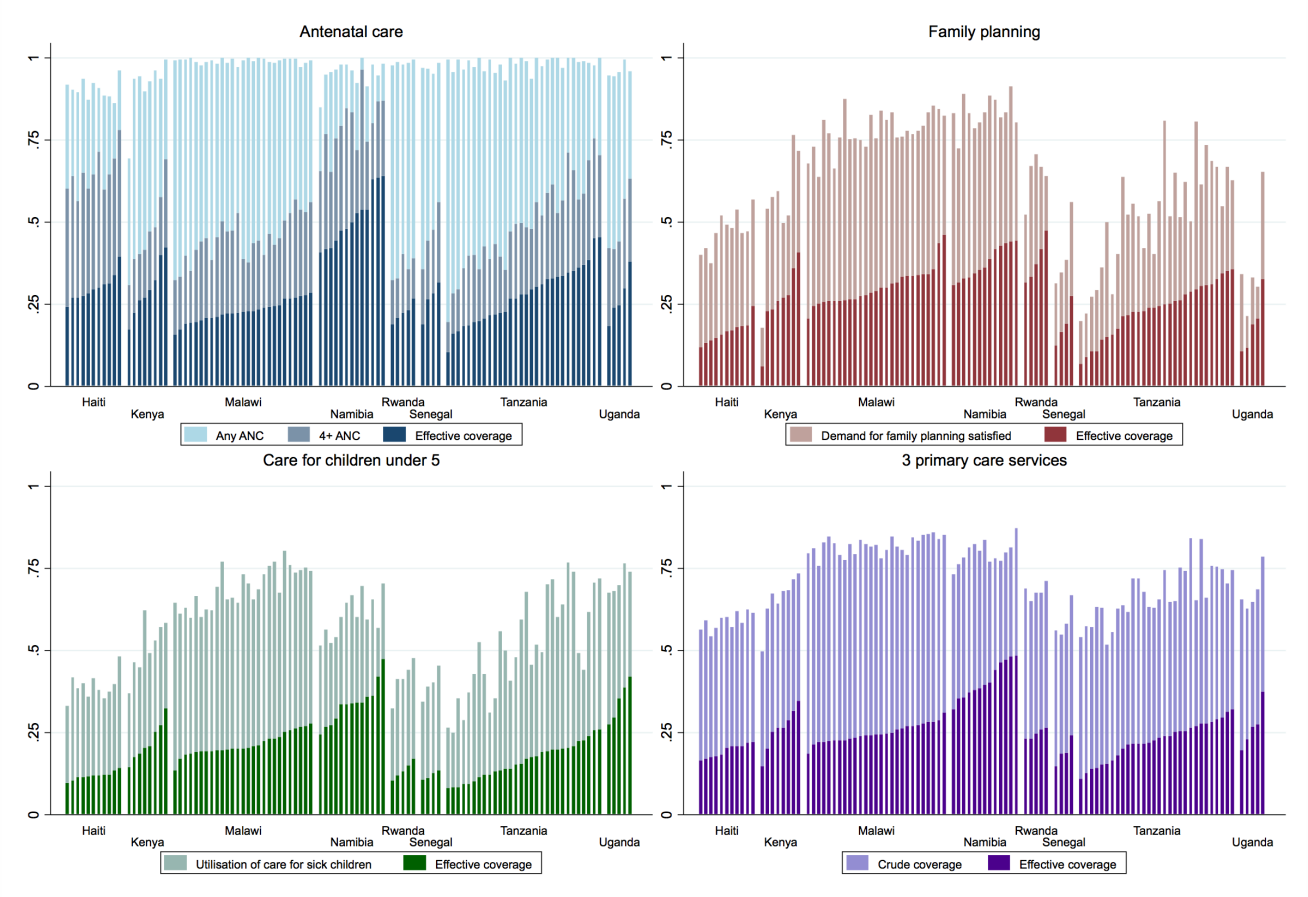
Crude and effective coverage of primary care services within the eight countries (n=103 regions). ANC, antenatal care.

Within these eight countries, population and health system surveys could be summarised into 103 unique subnational regions, ranging from 4 regions in Senegal to 30 in Tanzania (see online supplementary table 2 for details). Figure 2 displays crude coverage and effective coverage by subnational region for each service and the three-service aggregate. Across all regions and services, crude coverage substantially overstated population effective coverage: regions with effective coverage at least half as high as crude coverage were the exception rather than the norm. Differences between countries explained much of the variation in effective coverage, particularly in sick-child care (ICC 0.79, see online supplementary table 3). However, within each country, best-performing regions provided between 13% (sick-child care) and 20% (ANC and family planning) greater effective coverage than the worst regions. Within country, regions are ordered in increasing order of effective coverage, revealing particular quality deficits where higher crude coverage did not translate into higher effective coverage. Clinical quality was significantly correlated across services at the subnational level, ranging from a correlation of 37% for family planning and sick-child care to 61% for ANC and sick-child care.

**Figure 3:**
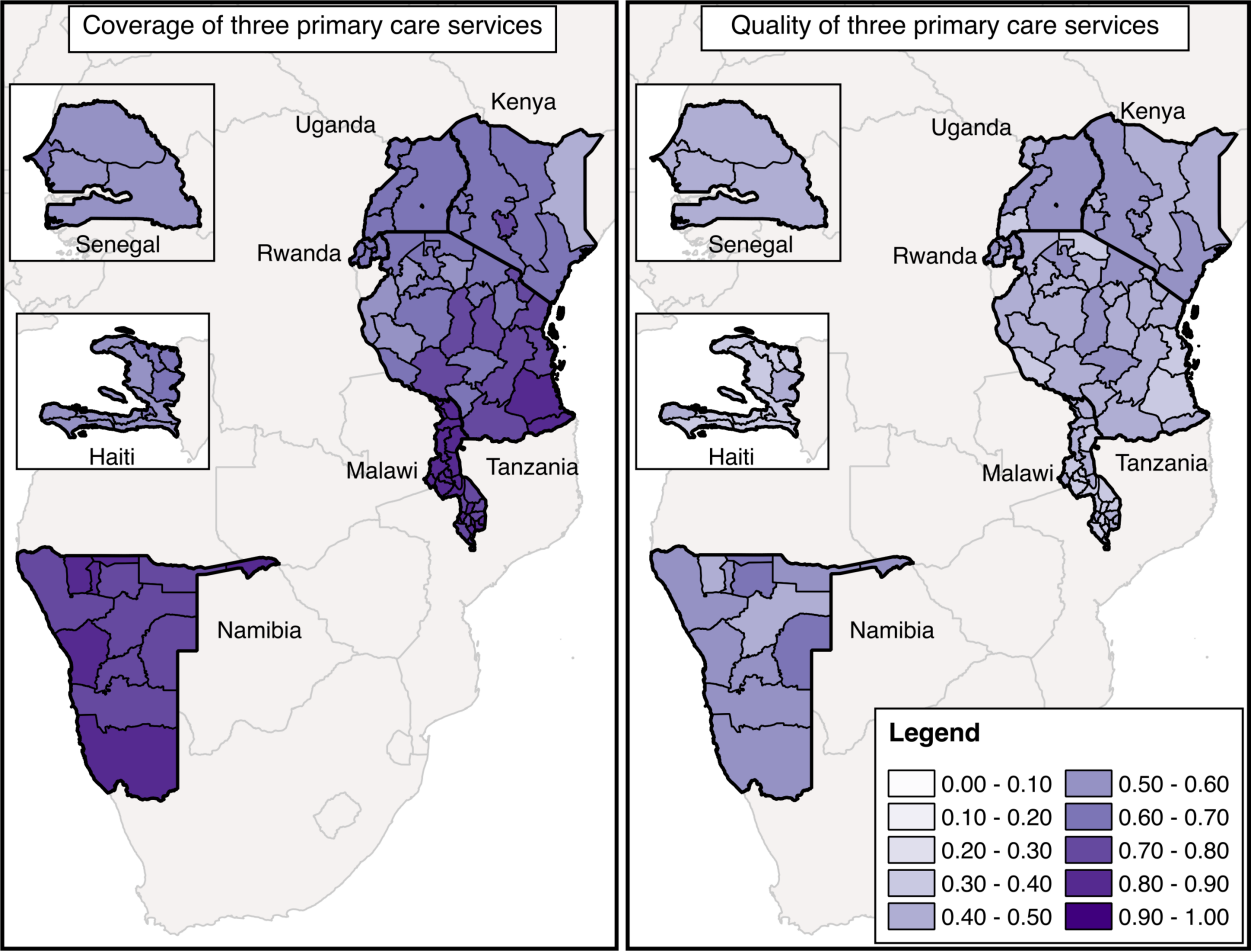
Coverage of primary care services and quality of primary care services in eight countries.

The maps of aggregate access and quality in figure 3 support the main findings above: quality lags access in every subnational region, and areas with better access do not necessarily provide better quality care. In fact, quality and use of care were significantly correlated only for quality of ANC and completion of four ANC visits (correlation of 21%, p<0.05).

## Discussion

This study is among the first to develop and apply metrics of effective coverage to multiple services in a crosscountry comparison using direct clinical observation to measure care quality. We found that in these eight countries, clinical quality lagged coverage and undermined effective delivery of all three essential health services. Three of every four women of reproductive age and children under 5 in need of health services did not receive highly effective services. Analysis of data from direct clinical observations of care showed care for sick children as the service with the lowest quality: in five of the eight nations assessed, healthcare workers performed fewer than one-third of basic clinical actions in visits with sick children.

Comparing the three services across countries provides insight into health system challenges and indicates potential priorities for improvement. While quality and access are both critical to effective coverage, our findings indicate that for ANC services, improving quality should be the main concern for all countries. Better quality is also the greatest challenge in countries such as Malawi and Namibia in family planning and possibly Uganda and Malawi in care for sick children. The quality gap in Malawi is particularly striking, with average crude coverage of over 80% but quality of only 0.4 out of 1 across the three services. High coverage within each service attests to the coordinated efforts to increase healthcare access for women and children in Malawi over the past decade despite limited resources.^31^Healthcare coverage in Malawi equalled that in Namibia despite the nearly 20-fold difference in healthcare expenditure between these countries. Clearly the quality of care delivered is now an urgent priority in order to deliver the expected health gains.

Improving quality is likely to increase effective coverage indirectly as well as directly: multiple studies have found associations between quality of care and subsequent utilisation. Research in Tanzania and Nepal found that women who received more services early in ANC were more likely to remain in care throughout pregnancy^32 33^; women’s perception of health facility quality was associated with utilisation of both maternal and child health services in a small study in Kenya.^34^ Our finding of a relationship between access and quality at the subnational level for complete ANC alone supports this link.

In working towards improved effective coverage, variation in access and quality within countries can help policymakers to identify the better performing regions that can provide guidance on best practices. The correlation of quality across these three primary care services suggests both that the lowest performing regions should be targeted for comprehensive quality improvement and that successful integration of high-quality services could yield benefits across maternal and child health services. Given the consistently low performance on basic clinical guidelines, however, quality improvement strategies that encompass all facilities and healthcare workers in the country should be considered.

Our findings of poor quality and a dramatic gap between crude and effective coverage align with existing literature in this field, with multiple studies in ANC in particular documenting low receipt of essential services despite differences in quality metrics and data source.^15–23^ The largest such study found that in 32 of 41 countries, over 95% of women received incomplete ANC based on a simple eight-item index.^19^ Although much of the research on ANC quality and effective coverage depends on accurate recall by women pregnant up to 5 years prior to the interview, we calculated quality based on directly observed clinical encounters, eliminating recall error as an explanation for low-quality care. Our work differs from most prior estimates of effective coverage of ANC in considering clinical interventions such as testing and prescriptions, and patient history and counselling elements that may be harder to assess from patient recall but are critical components of comprehensive ANC. For all services, we calculated effective coverage using average adherence to international guidelines on the premise that each element of the international guidelines contributes to better health outcomes.

Relatively little research exists on effective coverage of family planning and sick-child care. While past research has documented poor quality of clinical care from sources such as SPA surveys,^35–37^ to our knowledge, this study is the first to calculate effective coverage of these services using observed measures of clinical quality. Other effective coverage studies have used input measures such as facility type or readiness based on infrastructure and supplies.^8 16^ Using more proximal measures of effectiveness of healthcare delivery, we find equal or greater deficits in effective coverage than prior research.^16^ Our results support and broaden a prior study on effective coverage of malaria treatment, which similarly identified higher effective coverage for children in Uganda than most other sub-Saharan African countries.^24^ As a whole, the results emphasise the inadequacy of care delivered in this cross-section of low-income and middle-income countries, even for services that have long been global health priorities. Effective coverage for sick children averaged 12%–35% despite coordinated international efforts to improve child health through the Integrated Management of Childhood Illness programme, for example.^38^ The findings also suggest that efficiency gains are possible, as countries at very different levels of health spending demonstrated comparable levels of coverage and quality.

Beyond the insights permitted by comparing the three services, differences among the services and their measurement should be considered in interpreting the results. In ANC, a single visit indicates minimum necessary service utilisation while four visits reflects the number of visits recommended to achieve the intended content of care and repeated monitoring at the time of these surveys^28^ (since updated to a recommendation of eight visits^29^). Our results strengthen the conclusion of prior work that simply attending four visits is an inadequate proxy for receiving complete care.^19^ As measurement evolves to reflect the revised guidelines, consideration of the appropriate timing and total content of care during pregnancy should be privileged over the number of visits. For family planning, we selected met need for modern contraception as the coverage indicator in keeping with global monitoring efforts. This indicator reflects access to care and receipt of a commodity, a more proximal indicator to the ultimate outcome than care seeking alone. To this metric we add consideration of quality of care, which is strongly associated with the long-term and consistent use of family planning.^39–44^ The resulting effective coverage figures may be more conservative estimates than those for ANC and sick-child care. Finally, the measure of quality for sick-child care reflects provider adherence to good clinical practice; even providers adhering to guidelines have been found to diagnose or treat incorrectly,^45 46^ suggesting the low estimates of effective coverage in this study may still overstate children’s expected health benefits from seeking care.

There are several limitations to this work. The data sources spanned 2007–2016 and include countries at a wide range of income levels, limiting contemporaneous crosscountry comparisons. Linking population and facility surveys to calculate effective coverage raises important analytic issues,^47^ notably the difficulty in matching the sources of care sought with the quality of care received. Facility-based estimates of healthcare quality may not fully reflect use patterns due to lack of complete information on caseload and the exclusion of extensions beyond facilities such as community health workers and mass immunisation or supplementation campaigns.^48^ We linked utilisation metrics to quality at the subnational level—for example, districts, provinces—which prevents us from assessing the lower level variation in access and quality that is likely most relevant to communities and families. The small sample size at the country level and lack of subnational data on health expenditure preclude detailed analysis of cost-efficiency in achieving effective coverage. The regions used for sampling in these surveys did not always align between surveys or with national boundaries; some regions may include considerable internal variation that we are unable to estimate. Changes over time may have introduced error in the results, particularly for countries such as Namibia and Rwanda, with the greatest elapsed time (up to 4 years) between population and health facility surveys, if either quality or coverage changed substantially. Although population and health facility surveys provide the best source of data for these countries, where vital registration and health management information systems remain incomplete, these surveys are infrequent and planned separately, making extrapolation over time necessary in many cases. It is difficult to incorporate all sources of uncertainty—including in population size estimates as well as in bridging health system measures and actual population experience of care—in effective coverage estimates. Timely and locally specific data on healthcare need, utilisation and quality are critically needed to enable more granular understanding of effective coverage and research on its determinants at the local, regional and national levels. Looking forward, there is a need for better coordination of data sources on need, coverage and quality, as well as greater consensus on metrics of care quality. Future efforts should consider the previously unaddressed question of patient experience as an element of truly effective coverage.

This study is among the broadest assessments of effective coverage to date, with assessment of three primary care services across eight countries. It relies on direct observation of clinical care, which is not subject to recall error and provides more complete data than routine documentation.^49^ While direct observation can result in overestimates of quality due to the Hawthorne effect,^50^ the impact of the Hawthorne effect and any observer error are diluted by the large sample sizes and repeated observations of each provider within the SPA surveys. The SPA surveys are nationally representative; data for four of eight countries included are based on nearly complete census of the health system. Unlike many sources of health system data, SPA surveys include both public and private facilities. Observations conducted during the assessment provide a representative sample of health system users for the entire country, providing a robust source of evidence for the quality of care as it is delivered to those actually using it, in contrast to quality metrics based on population samples. Household studies provide the most representative sample for unbiased estimates of health system utilisation.

The MDG era saw rapid improvements in health system coverage; reaching the SDGs will require much stronger emphasis on effective coverage. With effective coverage emerging as a critical measure of progress towards universal health coverage, there is a growing need for comparative, evidence-based metrics. We demonstrated the potential to quantify quality in three primary care services and to calculate comparable effective coverage estimates at the national and subnational levels. The magnitude of the quality deficits identified in the study countries calls for urgent attention to improving the quality of essential health services alongside efforts to ensure women and children reach healthcare.
