# Supplementary material for: Effective coverage of primary care services in eight high-mortality countries
Source: BMJ Glob Health. 2017 Sep 4;2(3):e000424. doi: 10.1136/bmjgh-2017-000424 (PMC5887868; doi:10.1136/bmjgh-2017-000424)
Supplement: Supplementary file 1 [file bmjgh-2017-000424supp001.pdf]

Supplement table 1: Quality indices for direct observation of clinical care

**ANTENATAL CARE**<br>*Quality defined as the percent of the following 21 or 22 items performed. Items recommended once or twice during three follow-up visits are down-weighted by one third and two thirds respectively*	First visit	Follow-up visit
**History Taking - Provider asked or checked:**		
Last menstrual period to calculate gestational age	X	
Prior pregnancy experience, e.g. stillbirth, neonatal death, heavy bleeding, assisted delivery, multiples, and spontaneous or assisted abortion	X	
Danger signs in current pregnancy, e.g. bleeding, fever, headache or blurred vision, swollen face or hands, tiredness or breathlessness, fetal movements	X	X
Previous complications on record		X
**Routine Examination – Provider performed, examined for or assessed:**		
Weight	X	X
Fundal height	X	X
Edema	X	X
Vaginal exam	X	
Blood pressure	X	X
Fetal heart rate		X
Ultrasound	X	
**Screening – Provider asked about, performed, or referred patient for:**		
Syphilis test	X	1/3
HIV test	X	1/3*
Anemia test	X	2/3
Blood group test	X	
Urine test (proteinuria, bacteriuria)	X	2/3
**Preventive measures**		
Prescribed or gave iron or folic acid or both	X	X
Provider prescribed or gave tetanus toxoid injection	X	1/3
Prescribed or gave intermittent preventive treatment in pregnancy (IPTp)		X
**Education – Provider counseled on:**		
Nutrition	X	X
Sleeping under an insecticide-treated net	X	X
Delivery planning: preparation (money, transport) and location	X	X
Emergency planning: supplies for home delivery	X	X
Breastfeeding		2/3
Post-partum and postnatal care		2/3
Pregnancy spacing		2/3
**Record keeping**		
Provider completed ANC card	X	X

*HIV retesting is recommended in countries with generalized HIV epidemics (>1% prevalence among women seeking ANC); this item was included in calculating quality for all countries except for Senegal.

FAMILY PLANNING

Quality defined as the percent of the following 16 items performed:

Reproductive history

Age

Living children

Last delivery date

Pregnancy complications

Last menstrual period

Desire for child / more children

Desired timing for birth of next child

Breastfeeding

Menses

Health history / exam

Blood pressure

Weight

Smoking

STI symptoms

Chronic illness

Pelvic exam

Counsel on method

Any counseling on method

SICK-CHILD CARE

Quality defined as the percent of the following 16 or 21 items performed:

	Children < 2 months	Children 2 months – 59 months
**History taking**		
Inability to drink anything	X	X
Normal feeding pattern	X	
Sick feeding pattern	X	
Cough or difficult breathing		X
Diarrhea and blood in stool (dysentery)	X	X
Fever		X
Vomiting		X
Convulsions	X	X
Maternal HIV status	X	X
Ear problems		X
**Routine examination**		
Weight	X	X
Plotted weight on chart	X	X
Temperature	X	X
Pallor		X
Edema of feet		X
Mouth (thrush in IMCI)	X	
**Drug administration and immunization**		
Checked immunization card or immunized	X	X
Vitamin A dosage	X	X
Deworming medication		X
**Client Education and Counseling**		
Explained how to administer prescribed medication	X	X
Directions for feeding	X	X
Described danger signs requiring return to facility	X	X
Scheduled/discussed return visit	X	X
Gave diagnosis		X

Supplement table 2: Description of common geographic regions in health system and population surveys

Country	N	Type	Notes	Population in 1000s Median (IQR)	Area in 1000 km^2 Median (IQR)
Haiti	11	Departments	Facility location from SPA data combined with administrative boundaries to separate Aire Metropolitaine as distinct region; household data from post-earthquake camps excluded from study	542.3 (326.6, 1040.9)	2.1 (1.6, 3.5)
Kenya	8	Provinces	National administrative boundaries preceding the 2010 modification	4276.2 (2757.6, 6146.4)	50.1 (10.9, 141.8)
Malawi	27	Districts	Cities sampled separately in MICS were re-grouped with surrounding district to match administrative boundaries; MICS was not conducted in Likoma district	367.2 (239.0, 606.3)	3.1 (1.8, 6.5)
Namibia	13	Regions	National administrative boundaries preceding the 2013 modification	126.8 (65.2, 192.1)	49.1 (26.6, 104.7)
Rwanda	5	Provinces	Four provinces and the City of Kigali	1937.3 (1792.2, 2368.7)	5.9 (3.3, 5.9)
Senegal	4	Geographic zones	The 14 provinces of Senegal were grouped into four geographic zones for these surveys.	2377.6 (1865.7, 3205.0)	50.8 (17.7, 80.5)
Tanzania	30	Regions	National administrative (regional) boundaries	1442.6 (882.8, 2036.1)	28.9 (17.1, 46.9)
Uganda	5	Study-specific areas	Study-specific boundaries for the household and health system surveys necessitated combining multiple areas in the central and eastern regions to create 1 completely overlapping region unique to this study.	3458.9 (2250.7, 3941.3)	21.8 (15.8, 33.4)

Supplement Table 3: Variation in coverage and quality within versus between countries (N=103 regions in 8 countries)

	Intraclass correlation	Intraclass correlation limiting to 5th – 95th percentile of regions
**Coverage**		
ANC – any	0.46	0.44
ANC – 4 visits	0.62	0.62
Demand satisfied for modern contraception	0.66	0.69
Under-5 care seeking	0.62	0.64
Average of three primary care services	0.68	0.68
**Quality**		
ANC	0.57	0.59
Family planning	0.48	0.47
Sick-child care	0.74	0.77
Average of three primary care services	0.60	0.64
**Effective coverage**		
ANC	0.63	0.66
Family planning	0.58	0.61
Sick-child care	0.79	0.82
Average of three primary care services	0.67	0.69
